# *PEERNaija*: A Gamified mHealth Behavioral Intervention to Improve Adherence to Antiretroviral Treatment Among Adolescents and Young Adults in Nigeria

**DOI:** 10.3389/frph.2021.656507

**Published:** 2021-07-30

**Authors:** Aima A. Ahonkhai, Leslie J. Pierce, Samuel Mbugua, Benjamin Wasula, Samuel Owino, Ashley Nmoh, Ifeoma Idigbe, Oliver Ezechi, Sandra Amaral, Agatha David, Prosper Okonkwo, Nadia Dowshen, Martin C. Were

**Affiliations:** 1 Department of Medicine, Infectious Diseases, Vanderbilt University Medical Center, Nashville, TN, United States; 2 Vanderbilt Institute for Global Health, Vanderbilt University Medical Center, Nashville, TN, United States; 3 Department of Medicine, Vanderbilt University Medical Center, Nashville, TN, United States; 4 Institute of Biomedical Informatics, Moi University, Kesses, Kenya; 5 Department of Medicine Health and Society, Vanderbilt University, Nashville, TN, United States; 6 Nigerian Institute of Medical Research, Lagos, Nigeria; 7 Department of Pediatrics, Children’s Hospital of Philadelphia, Philadelphia, PA, United States; 8 Center for Clinical Epidemiology and Biostatistics, University of Pennsylvania, Philadelphia, PA, United States; 9 APIN Public Health Initiatives (APIN), Abuja, Nigeria; 10 Craig-Dalsimer Division of Adolescent Medicine, Children’s Hospital of Philadelphia, Philadelphia, PA, United States; 11 Perelman School of Medicine, University of Pennsylvania, Philadelphia, PA, United States; 12 Department of Biomedical Informatics, Vanderbilt University Medical Center, Nashville, TN, United States

**Keywords:** mobile health, gamification, low- and middle-income countries, adolescents and young adult, medication adherence, HIV, social support

## Abstract

**Background:**

HIV is the leading cause of death for youth in Sub-Saharan Africa (SSA). The rapid proliferation of smart phones in SSA provides an opportunity to leverage novel approaches to promote adherence to life-saving antiretroviral therapy (ART) for adolescents and young adults living with HIV (AYA-HIV) that go beyond simple medication reminders.

**Methods:**

Guided by the Integrate, Design, Assess and Share (IDEAS) framework, our multidisciplinary team developed a peer-based mHealth ART adherence intervention—*PEERNaija.* Grounded in Social Cognitive Theory, and principles of contingency management and supportive accountability, *PEERNaija* delivers a multi-faceted behavioral intervention within a smartphone application to address important obstacles to adherence.

**Results:**

*PEERNaija* was developed as a gamified Android-based mHealth application to support the behavioral change goal of improving ART adherence among AYA-HIV within Nigeria, a low- and middle- income country (LMIC). Identified via foundational interviews with the target population and review of the literature, key individual (forgetfulness and poor executive functioning), environmental (poor social support) and structural (indirect cost of clinic-based interventions) barriers to ART adherence for AYA-HIV informed application features. Further informed by established behavioral theories and principles, the intervention aimed to improve self-efficacy and self-regulation of AYA-HIV, leverage peer relationships among AYA to incentivize medication adherence (via contingency management, social accountability), provide peer social support through an app-based chat group, and allow for outreach of the provider team through the incorporation of a provider application. Gamification mechanics incorporated within *PEERNaija* include: points, progress bar, leaderboard with levels, achievements, badges, avatars and targeted behavior change messages. *PEERNaija* was designed as a tethered mobile personal health record application, sharing data to the widely deployed OpenMRS electronic health record application. It also uses the secure opensource Nakama gamification platform, in line with *Principles of Digital Development* that emphasize use of opensource systems within LMICs.

**Conclusions:**

Theory-based gamified mHealth applications that incorporate social incentives have the potential to improve adherence to AYA-HIV. Ongoing evaluations of *PEERNaija* will provide important data for the potential role for a gamified, smartphones application to deliver multifaceted adherence interventions for vulnerable AYA-HIV in SSA.

## INTRODUCTION

Antiretroviral therapy (ART) scale-up has led to unprecedented progress in the global AIDS response, but these gains have not fully benefited the four million adolescents and young adults (15–24 years) living with HIV (AYA-HIV) (1). This is especially true in Sub-Saharan Africa (SSA) where 85% of the world’s AYA-HIV reside (1–3). Excellent adherence to ART (~ >80–90%) is required to effectively treat HIV infection (4–6). However, as few as 40% of AYA-HIV who have started ART are adherent (1, 7). As a result, AYA-HIV show unacceptably high rates of virologic failure (30–50%) (4–6, 8). Poor adherence to life-saving ART is an important contributor to morbidity and mortality from HIV. Over the past decade, while HIV-related deaths decreased by 30% overall, HIV-related deaths among AYA increased by 50%, making HIV the leading cause of death among this population (1–3). These data underscore the urgent need to identify and evaluate interventions to improve medication adherence and HIV outcomes for AYA-HIV in SSA.

Studies of AYA living with HIV in SSA have identified a range of individual, environmental, structural, and treatment-related factors that impede medication adherence (9). At the individual level, “forgetting to take the dose” is identified as an adherence barrier in approximately 63% of patients (9). The seemingly simple act of forgetting may be driven by cognitive factors that result from complications of HIV infection and/or age-specific developmental factors (9). Youth with perinatal HIV infection, even when it is optimally treated, are at increased risk for cognitive dysfunction that may impede self-efficacy for disease management (10). In addition, adolescence itself is characterized by a developmental mismatch between limbic and pre-frontal cognitive functions leading to impulsivity, risk-taking, and poor concrete thinking that may also challenge the behaviors necessary to remain adherent to chronic medical therapies such as ART (5, 8, 11). Not surprisingly, being asleep (37%) or busy (25%) are also commonly cited as reasons for missing ART doses, but may also reflect the difficulty of organizing one’s schedule and priorities to include disease management (10). AYA-HIV also describe a number of treatment-related barriers to adherence including pill burden, palatability, and fear of side effects which can unfortunately worsen if they are prescribed more complicated regimen to treat drug-resistant HIV that develops in response to poor adherence (5, 10).

Stigma–described as a discrediting “mark” that reduces the status of the person in the eyes of society–has been identified as a barrier to adherence among 40% of youth (10). Stigma may often push AYA-HIV into isolation with their disease and ultimately impede adherence to HIV care and treatment (12, 13). Social support is a critical environmental factor that can help to combat stigma and isolation for AYA-HIV (5, 12–15). Structural barriers to medication adherence identified by AYA-HIV include indirect costs of care (cost of travel to the clinic or pharmacy, lost wages incurred on clinical or drug pick-up days for the patient, and caregiver, etc.) (40%) (16, 17). These barriers may pose unique challenges for AYA-HIV who are often not fully autonomous nor financially self-sufficient and dependent on others for these resources (5).

The use of digital health solutions, especially medication reminders delivered via mobile health (mHealth) platforms, have shown promise as adherence support tools for people living with HIV in SSA (18–24). mHealth solutions are uniquely suited to tackle a range of psychosocial barriers by exploiting an ecological momentary approach (EMA), a framework for assessing behavior and delivering interventions to people as they go about their daily lives, in real-time and in real-world settings (25). However, most existing mHealth interventions for medication adherence that target patients within low- and middle- income countries (LMICs) rely solely on simple short message service (SMS)-based approaches (22, 26, 27). SMS reminders alone may not be sufficient to improve medication adherence for many AYA-HIV. The complex nature of the barriers to medication adherence for AYA-HIV suggest that successful adherence interventions may need to adopt a multi-faceted approach (27). Accordingly, a recent systematic review of adherence interventions for AYA-HIV in LMICs concluded that future interventions need to address broader psychosocial barriers for this vulnerable population (28). Few studies have investigated novel approaches such as the use of gamification, social incentives, and EMA interventions within smartphone-based behavioral interventions (29, 30). With rapid and exponential growth in usage of affordable smartphones in LMICs, and AYAs in these settings being early adopters of communication technologies, there is a unique opportunity to implement such innovative approaches (31).

In this paper, we describe the design and development of the *PEERNaija* smartphone application. Informed by *TreatYourSelf*, a smart phone-based application designed with input from AYA-HIV in Philadelphia, was created using an EMA for AYA-HIV in the United States (US), and has demonstrated feasibility and short-term (3 month) improvement in self-reported adherence (32–34). Key features of the guiding application include medication reminders and adherence tracking, refill and appointment reminders, leaderboard and adherence points, internal community supports through discussion forums, peer-to-peer kudos, and community-based resources list. Similarly, *PEERNaija* is a theory-driven and user-centered gamified smartphone application designed to improve medication adherence for AYA-HIV living in LMICs, with the application’s initial implementation focused on Nigeria. We report on *PEERNaija*’s development approach, the behavior change techniques incorporated, and the application’s features and functionalities with emphasis on implemented gamification mechanics and social incentive features.

## MATERIALS AND METHODS

### Objective

This project set out to develop a novel mHealth peer-based intervention that utilizes key behavioral interventions within a smartphone application to promote medication adherence for AYA-HIV living in LMICs. In addition to medication reminders, the proposed intervention aims to provide social support, integrate gamification strategies, and leverage the currency of social incentives to promote medication adherence among AYA-HIV.

### Setting and Participants

The proposed solution targets AYA-HIV patients living in LMICs, with particular emphasis on AYA-HIV who have poor adherence to their ART medication. As a smartphone-based solution, users need to own or have access to a smartphone device. The initial implementation setting for the application was in Nigeria. Nigeria has had the highest annual incidence of perinatally infected children for the past seven years; 26.9% of all perinatally infected children worldwide (5, 35). Coupled with ongoing behavioral transmission, Nigeria, Africa’s most populous nation, is home to 10% of the global population of AYA-HIV (36, 37). The target implementation site was the Nigerian Institute of Medical Research (NIMR) in Lagos. With a population of more than 13 million, Lagos is Nigeria’s biggest city and one of the President’s Emergency Plan for AIDS Relief (PEPFAR) priority states for effective ART scaleup (38). NIMR began providing HIV care in 2002 with support from the PEPFAR program. As of December 2020, NIMR had nearly 300 AYA enrolled in care.

### *PEERNaija* Development Approach

**FIGURE 1 |:**
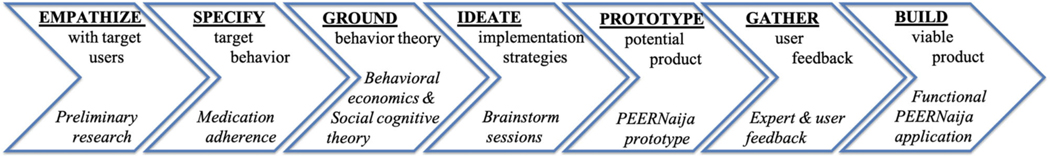
Seven steps of the IDEAS framework employed in the development of *PEERNaija*.

The Integrate, Design, Assess and Share (IDEAS) framework was used to develop *PEERNaija*. IDEAS is a theory-based framework for designing and testing digital behavioral health interventions successfully employed in the US for patient-facing applications (39, 40). IDEAS is comprised of multiple steps, namely: empathize, specify, ground, ideate, prototype, gather, build, pilot, evaluate, and share (41). The development of *PEERNaija* as described in this paper involves the first seven steps, which focus on application development (Figure 1) (41). As outlined by Mummah et al. these seven steps can be described as follows:

“*Empathize:* gather qualitative insights from users (e.g., in-depth interviews, focus groups). *Specify:* translate broad behavioral goals into a highly specific target behavior, taking into consideration actionability, health impact, and user acceptability. *Ground:* ground intervention in behavioral theory and evidence and incorporate relevant behavioral strategies. *Ideate:* brainstorm creative strategies for translating theory and user insights into features, using inspiration from wide and varied sources. *Prototype:* develop rough prototypes of ideas rapidly and iteratively, sharing, discussing, and improving prototypes as a cross-sector team. *Gather:* gather user feedback on prototypes (e.g., interviews, questionnaires), and uncover insights to inform product improvement. *Build:* build a fully functional minimum viable product, and incorporate app analytics to collect data on app usage patterns (40).”

Due to constraints placed on in-person gatherings by the COVID-19 pandemic, the initial *Gather* phase relied on several key stakeholders, but not on end-users. The study team decided to proceed with stakeholder feedback to build the fully functional minimum viable app from which to gather detailed user feedback once in-person meetings were again permitted in Nigeria.

## RESULTS

Below, we report details of the *PEERNaija* development process, as well as the resulting product based on the IDEAS phases, with each phase used to inform the next step of the development process.

### Phase 1: Empathize With Users

Research by Ahonkhai et al. with AYA-HIV in Nigeria, experience of Dowshen et al. in the development of the *TreatYourSelf* application (32–34) for AYA-HIV in the US, and data from systematic reviews highlighting barriers to medication adherence guided the *Empathize* phase of the development process (4–6, 8). Dr. Ahonkhai’s preliminary quantitative data demonstrated that 40% of Nigerian AYA-HIV who remained in care 1 year after starting ART had virologic failure (HIV RNA > 1,000 copies/mL) (4). Further, in a multi-site study of adherence measured by medication possession ratio (MPR), which is the proportion of prescribed doses of ART picked up from the pharmacy, AYA-HIV with MPR > 94% had a marked reduction in the risk of virologic failure in the first year on ART compared to those with MPR < 80% (aRR 0.43, *p* < 0.001) (15). Nonetheless, 26% of AYA-HIV with optimal adherence by MPR (>94%) still had virologic failure, highlighting discordance between ART pick-up from the pharmacy and ART taking behavior among some youth (15). These data underscored both the high prevalence of virologic failure and the need for adherence support measures to help AYA-HIV to take their medicines.

Qualitative insights into the needs and thought processes of AYA-HIV were gathered from formative, in-depth interviews from Nigerian AYA-HIV, as well as by Dowshen et al. through the development of the *TreatYourSelf* mHealth application (32–34). Ahonkhai conducted semi-structured in-depth interviews with 20 AYA-HIV at the study site, NIMR. Among them, 95% (*n* = 19) owned mobile phones, and 65% (*n* = 13) owned smart phones. Only one participant reported that his/her phone was shared, and 90% (*n* = 18) owned their own devices (Ahonkhai, personal communication, 2019). Most of those surveyed were frequent users of SMS/text messaging (80%, *n* = 16), WhatsApp (65%, *n* = 13), and social media platforms (55%, *n* = 11) (Ahonkhai, personal communication, 2019). Most youth (65%, *n* = 13) were not fully confident in their ability to remember to take a daily medicine and relied on daily reminders of some sort (parent or alarm most common). Indeed, half (*n* = 10) also reported missing medications at least once in the past 2 weeks. Thematic analysis revealed that interviewed youth were highly engaged with their mobile phones and interested in potential mobile phone-based strategies to help with adherence. They had not been exposed to adherence apps themselves but thought app-based reminders and peer social support would be important features of such an intervention. Further, through the conduct of formative participatory design workshops, the *TreatYourSelf* team identified five themes that were important to users: (1) positive and non-judgmental tone, (2) minimal, avatar-based gamification, (3) motivational messages, (4) non-disclosure through neutral signifiers, and (5) social support through camaraderie (32–34). All but one (theme 2) of these themes were supported by growing literature on SMS-based reminder interventions (26, 29, 30) and was consistent with the overall sentiments of Nigerian youth; thus, were incorporated into the *PEERNaija* prototype (32–34).

Integrating these quantitative and qualitative research experiences in Nigeria and the US with reviews of the literature led us to focus on an intervention package that would help to overcome the issue of forgetting to take medication (9). The intervention package would also provide critical social support (5, 12–15), while being very mindful not to magnify other barriers such as stigma (12, 13), and time/transportation/resources to travel to the clinic (16, 17).

### Phase 2: Specify Target Behavior

We determined that the intervention would aim to target increased consumption of ART medication (or medication adherence) in AYA-HIV. Optimal adherence to ART is central to achieving the primary public health goal of HIV treatment—suppression of HIV replication below the limit of detection of commercially available assays (42). This goal is critical not only for the health of PLWH, but for the prevention of HIV transmission in the community (43). The level of medication adherence required to achieve viral suppression depends on a number of factors including the potency and durability of the ART regimen in addition to the amount of time on ART (44). Researchers have historically identified an adherence threshold of >94% as a gold standard for viral suppression, but more contemporary ART regimens may require less stringent adherence levels (80–90% or less) (44). However, the most contemporary regimens are still being scaled up in LMIC. Therefore, we will target traditional thresholds for ART adherence, namely optimal (>94%), suboptimal (80–94%), and poor (<80%) adherence based on existing literature associating with risk of virologic failure (45). Monthly adherence rates were calculated as follows: (number of doses reported within a 4-h window/number of doses ∗ 100%).

### Phase 3. Ground in Behavioral Theory

Our intervention approach is grounded in Bandura’s (46, 47) Social Cognitive Therapy (SCT), and the foundational behavioral economics principle of incentivization of human behaviors (48). SCT explains health behaviors via their dynamic and reciprocal interaction between individuals and their environments. SCT supposes that behavior change is informed by an individual’s knowledge, self-efficacy, outcome expectations (cost and benefit of health habits) and health goals (46, 47). Our intervention is designed to address both self-efficacy and outcome expectations. Medication reminders promote self- efficacy by enhancing self-regulation, while also overcoming cognitive barriers to adherence (e.g., forgetfulness). Bandura’s SCT has been expanded to incorporate interactive media, such as digital health interventions, that exploit a key principle—that individuals learn from both direct experiences and social/observational modeling (by watching others) (46, 47). In this way, observing others who resemble us achieve desired outcomes can create outcome expectations. In addition to creating opportunities for social/observational modeling, mHealth interventions offer a unique form of interaction, motivation, and accountability for patients. In 2011, Mohr et al. developed a conceptual model, known as Supportive Accountability, to further characterize the relationship between adherence to mHealth interventions and human support using principles from organizational psychology, motivation theory, and computer-mediated communication (48). Mohr proposed that adherence behaviors are impacted by human support factors driven by bonds, accountability, and legitimacy of support and are moderated by both patient motivation and form of mHealth communication (48).

We have also incorporated additional behavioral economics principles that directly address outcome expectations through the use of peer-based social incentives. Attitudes and behaviors of AYA are strongly influenced by their peers (49, 50). Early research has primarily focused on the link between peer influence and undesirable behaviors, which are sometimes adopted and spread via social networks through a process coined social contagion (51, 52). Several studies however, highlight the important role of peer relationships on a range of desirable health and social behaviors (e.g., volunteerism) (53, 54). Peer relationships may influence behaviors through the exertion of descriptive norms (what is most commonly done, or what individuals perceive to be so) and injunctive norms (what ought to be done) for certain behaviors (55, 56). As such, peer acceptance and alignment with normative behavior may be a powerful social incentive to promote health behaviors in young people. Interventions have taken advantage of the power of social norms within AYA peer groups to address unhealthy behaviors such as excess alcohol consumption (57). Social incentivization is an important example of behavioral economics principles of contingency management. Incentives have been used widely to reward desired health behaviors (58). Conditional incentives for HIV treatment have been effective in small studies, with absolute increases in adherence ranging from 15 to 25% (59– 61). Similar data are few in LMICs, and even fewer on the role of peer-driven social incentives, a highly innovative aspect of our intervention.

### Phase 4. Ideate Creative Implementation Strategies

Based on insights gained from the intended target behavior (Phase 2) and the identified behavioral strategies to employ (Phase 3), the team comprised of experts in adolescent medicine, HIV medicine, epidemiology and implementation research, behavioral health, bioinformatics, and game design engaged in several group brainstorming sessions to *Ideate* on the proposed solution. This phase also included the software development team that was well-versed with product development. Requirement analysis revealed the need for an application focused on the following key features, namely: (a) provision of daily medication reminders, (b) recording of medication doses taken, (c) tracking of medication adherence in real-time, (d) comparing adherence performance of individuals relative to their peers, and with the desired normative behavior (social incentive), and (e) provision of social support through peer communication features.

Given that *PEERNaija* was to be used by patients to manage their care, it can be classified as a mobile personal health record (mPHR) application. PHRs are “*electronic applications used by patients to maintain and manage their health information in a private, secure, and confidential environment* (62).” Studies show that standalone PHRs, which are only used by individual patients without the data being shared to health professionals, are not as effective as when PHRs are tethered or connected to electronic health record system (EHRs) (tethered PHRs) (63–66). As such, it was decided that the P*EERNaija* application should be tethered to the opensource OpenMRS EHRs, which meant that patient-level data collected via *PEERNaija* app would be transmitted and availed to providers using the EHRs. OpenMRS was chosen given that it is the most widely-endorsed and used EHRs for HIV care in Nigeria and is also in use in over 50 other countries worldwide (67, 68). In addition, given the emphasis on open standards and open source applications for solutions within LMICs (as guided by the “*Principles of Digital Development*”) (69), we settled on the open source Nakama platform for the gamification components for the application (see Gamification server) (70).

Gamification is generally defined as the “use of game design elements in non-game contexts (71).” Gamification has been demonstrated to motivate behavior change and increase engagement in health care, and we utilized gamification strategies to deliver or enhance several core functions of the mPHR (72–75). Gamification strategies can influence behaviors by impacting both intrinsic and extrinsic motivation of individuals (76). Cugelman outlined seven core gamification ingredients that have clear linkages to behavior change strategies, namely: (1) Goal setting: commitment to achieving a goal; (2) Capacity to overcome challenges: growth, learning, development; (3) Providing feedback on performance: receiving constant feedback through experience; (4) Reinforcement: gaining rewards and avoiding punishments; (5) Compare progress: monitoring progress with self and others; (6) Social Connectivity: interacting with other people; and (7) Fun and playfulness: playing out an alternate reality (72). Others have additionally described immersion features such as avatars and virtual identity (77). Among the various gamification mechanics, leaderboards, points and badges make “the trinity” often seen as a base requirement in any gamified application (78). Which additional gamification mechanics to incorporate largely depends on their fit to support the targeted behavior change (77). Increasingly, research shows that incorporation of additional game elements leads to “great presence, enjoyment and effort [a proxy for motivation],” with most applications having an average of five gamification elements (79). Applying these ingredients within mHealth applications through the use of various gamification mechanics can motivate behavior change.

### Phase 5: Prototype Potential Products

The project team engaged a User Interface (UI) and User Experience (UX) expert to develop various prototypes for the *PEERNaija* application. For this, we used the Adobe XD UI/UX design collaboration tool, with resulting storyboard shared, discussed and iteratively refined by the team (80). Further, the back-end server-side features connecting to both OpenMRS and Nakama servers were developed and tested. All features that had been identified in *Phase 4: Ideate* were also implemented. Technical aspects of the *PEERNaija* app development were informed by the Technology Acceptance Model (TAM) (81). Derived from social psychological theories of reasonable action and planned behavior, TAM posits that perceived usefulness and ease of use of new technologies mediate behavior intention, use and acceptance of the app. TAM will be used to predict *PEERNaija* use through routine collection of user feedback (assessing a variety of concepts including ease of use and acceptability) at each study encounter, and assessment of paradata (81). We thus emphasized approaches to improve engagement and acceptance of the *PEERNaija* app, focusing on easier onboarding, push notifications, personalized dashboards, and product iteration.

### Phase 6: Gather User Feedback

We conducted two community engagement studio sessions to gather feedback on the first iteration of the PEERNaija application. Community engagement studios are dynamic, consultative sessions intended to elicit stakeholder input on the planning, design, implementation, translation, or dissemination of a program aimed at improving health related outcomes (82). The process is more deliberative than a focus group, and the participants are compensated as stakeholder “experts” (82). The first studio session included Children’s Hospital of Philadelphia researchers who developed the *TreatYourSelf* mobile application for use among AYA-HIV in the US (32–34). For the second studio session, we invited key stakeholders who work with AYA-HIV in Nigeria including: (a) public health officials active in the oversight and administration of a network of HIV care and treatment centers, (b) pediatric and adolescent providers, and (c) adolescent HIV counselors. User feedback from the target audience was delayed due to constraints of the COVID-19 pandemic. As a result, the study team made a strategic decision to gather detailed user feedback on the initial viable product (after step 7) so that the target users could be convened in person to view and provide feedback on a functional prototype. Given that the participants have not been exposed to apps of this type, it was felt that this approach would give the target users a more nuanced understanding of the app features for which they could provide rich feedback.

Stakeholder feedback from these studio sessions was analyzed using content analysis and included recommendations to (1) develop a provider-facing application to allow for monitoring of individual-level user adherence, (2) moderate chat room content by using peer champion, (3) encourage appropriate chat remove behavior by adding a pop-up notification that reminds users of community guidelines prior to entering the chat room and removing the ability for users to upload photos, and (4) ensure user-specific feedback on presentation, colors, and text in the graphical display.

### Phase 7: Build Minimum Viable Product

Based on user feedback, the developed prototype was further revised into a fully functional *PEERNaija* application. At this stage, several additional features were included, namely: (a) *Avatars* for use within the immersion feature, (b) logic to define reminders for medication, (c) on-boarding features to allow easy installation, setup and understanding of the application features, (d) application settings to allow changes in medication regimen, avatar, and user preferences on time to take medication. All gamification logic were also incorporated that defined how points accumulated within the application, rankings within leaderboards, and rules for fulfilling defined achievements within the application. Messages, information and feedback features were also programmed.

From feedback received, it was also deemed important to create an application for providers and program managers to give better insights into individual-level performance. While the providers could view clinical data through the EHRs, they needed to be able to see leaderboards, and to contact individuals privately through a chat feature who had poor adherence. These were incorporated within the provider application that allows providers to quickly identify patients who have not been adherent or engaged with the application.

Below, we provide details of the final viable *PEERNaija* product.

### *PEERNaija* APPLICATION

The application was called “*PEERNaija*” to highlight the social and peer-based nature of its features, as well as to capture the location of its initial deployment in Nigeria. Key implemented features within the PEERNaija app include: (a) reminders for AYA-HIV to take medication, (b) ability to record doses taken, (c) chat-based social feature, and (d) several gamification features and mechanics, including points, progress feedback, leaderboards, badges, and avatars. These features were incorporated to align with several behavioral change techniques targeted for AYA-HIV (Table 1).

### *PEERNaija* Application Architecture

**FIGURE 2 |:**
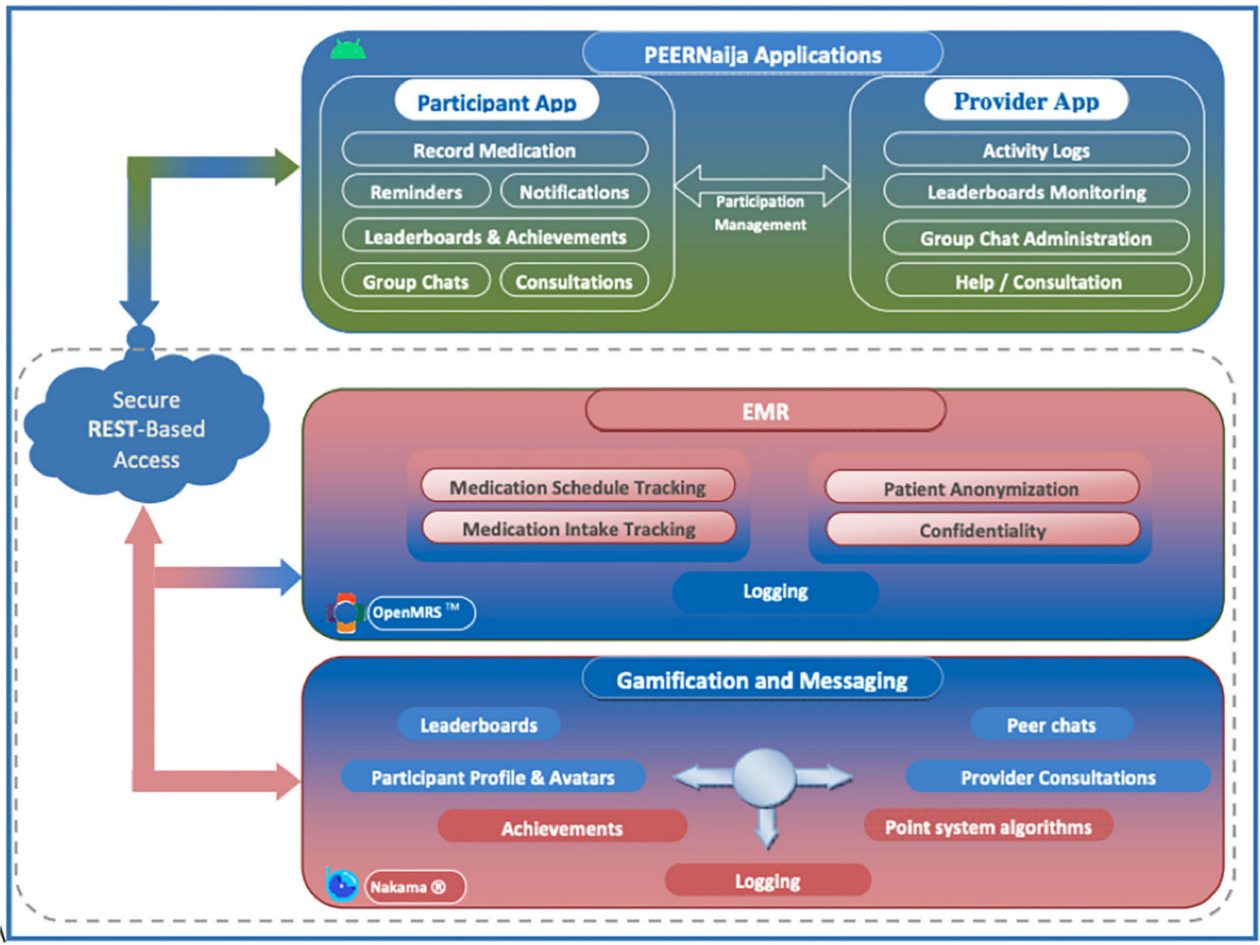
*PEERNaija* architecture.

Figure 2 provides the overall architecture of the developed *PEERNaija* application. The application has three main components including an electronic medical record system component, gamification server, and the smartphone-based mobile application.

Health information exchange between *PEERNaija* and OpenMRS is achieved through the use of terminologies, location, provider, and patient identifier information that are shared between the two systems, as well as a Representational State Transfer (REST)-application programming interface (API) (83). The clinical data collected through *PEERNaija* are synchronized back into OpenMRS in real-time or on-demand with the NigeriaEMR OpenMRS distribution (84). With this approach, *PEERNaija* avoids being a siloed system, with the data available for decision making to providers who have access to the linked OpenMRS implementation.

### EMR Interoperability

*PEERNaija* was developed as a tethered PHR (see Phase 4 above for additional description). Data collected includes medication regimen options and medication update records as entered by the patient on the mobile application. Data interface with the OpenMRS based EMRS is protected over a transport layer security (TSL) connection and with a firewall.

### Gamification Server

*PEERNaija* uses the open source Nakama game server for its gamification functionality (Apache 2.0 license) (70). The Nakama platform was chosen given its broad use and its inclusion of required gamification features, user account management and reliable messaging functionality. Unlike other options, Nakama allowed the project to install its own dedicated gamification server, with no data being shared to any third party, hence ensuring security and confidentiality. Nakama server is developed in GoLang, the Google supported programming language optimized for building gaming applications (85). For its backend database, Nakama utilizes CoackroachDB (86). Nakama ships with its own inbuilt web server simplifying its implementation and management.

### *PEERNaija* Mobile Application Description

*PEERNaija* was developed as Android application, recognizing that in Africa over 85% of smartphones use the Android operating system (87). On installation, the user is taken through several onboarding screens to familiarize them with how to use the application. They are then provided with the application *Terms & Conditions, Privacy Notice*, and *Community Guidelines* to which they have to agree. After this the user can then create an individualized profile, which includes: (a) selecting their preferred avatar and nickname for their immersion experience, (b) choosing their ART medication regimen from a pre-selected list (that can be validated by the provider), (c) selecting preferred times for medication reminders, and (d) creating a preferred reminder message, with several choices also availed (Figures 3.1–3.8). Profile features can be changed at any time through the Settings side-bar menu.

A username and password, which are initially authenticated against the EMRs, are required for access to the PEERNaija mobile application. Once the user profile is set, the user is taken to the home screen that will be the landing page for all subsequent logins (Figure 3.1). This home screen is a personalized dashboard that includes several features, namely: (a) *a welcome and notification area*–used to provide informative, evaluative and comparative feedback to the user in a culturally- and age-appropriate language, (b) *points display*–indicating the number of points earned by the individual from taking their medication, (c) *a progress bar* that indicates the individual medication adherence level, (d) *Recent medication doses taken*–a color-coded summary of the individual’s medication doses recorded for the week, (e) *rank*–a snapshot of the individual’s position in the leaderboard. From the home screen, users can access detailed information on their monthly intake (Figure 3.3), and of the full leaderboard (Figure 3.4). The application also contains a Tab bar menu at the bottom, that allows access to *Achievements*, the peer-based *Chat* functionality, and a *Consult* feature where the individual can contact a care provider privately and securely.

*PEERNaija* is programmed to always run in the background when the phone is on. Its key feature includes a notification to the AYA-HIV on when their next dose is due (Figure 3.5). Notifications are programmed to appear as reminder message within an hour of the medication being due, with subsequent reminder messages showing up to 4 h after the due period. How the notifications appear depend on preferences and functionality for notifications with the device in use.

### *PEERNaija* Gamification Features

#### Medication Reminders

Medication reminders are based on the preferred time for taking medications that had been selected by the patient during setup. Medication reminder messages are programmed to first appear 30-min before the scheduled medication was due. If no action is taken, the reminder appears again when the medication is due, and every hour up to 4 h after the medication due time. Reminder messages can be customized or selected from *PEERNaija* provided cultural- and age-appropriate reminder choices. Examples of these reminder messages are provided in Table 2.

#### Points

*PEERNaija* app users accumulate points based on taking and recording their medication within 4 h of the scheduled time. Value of points and activities deserving of points were determined with user input. If all daily medication doses are appropriately taken/recorded the user earns 10 points. However, no points are awarded if the user records the medication more than 4 h after the due time. To facilitate competition and to avoid discouraging individuals, point accumulation started afresh each month.

#### Leaderboards and Levels

Leaderboards and levels serve to provide extrinsic incentives (88), and as progress indicators that can motivate performance (89). Within *PEERNaija*, we settled on three leaderboard levels that signified top, medium and bottom tiers. Individuals are ranked based on the points accumulated and adherence rates for the month. An adherence score of above 94% is considered optimal, whereas a rate between 80 and 94% is considered suboptimal and a rate <80% is considered poor. Individuals with the same number of points are given the same rank and listed in the chronological order in which they achieved those points.

#### Progress Bar

The progress bar within *PEERNaija* was used as an indicator of the medication adherence and showed the number of points earned thus far. The progress bar resets on a monthly basis.

#### Achievements and Badges

Specific achievements can be unlocked in *PEERNaija* and are described in Table 2. On unlocking an achievement, the AYA-HIV received a badge and a congratulatory message. By their very nature, adherence-related achievements become unlocked in a stepwise fashion based on the number of days of perfect adherence. These achievements reset every month.

#### Avatars

*PEERNaija* users an select an avatar from a set of 180 culturally-sensitive and age-appropriate Avatars. These Avatars were selected after review by peer consultants. Display of the avatar for various users was randomized to reduce congregation toward the same avatars. For the purposes of the pilot implementation, once a user selects an Avatar, it is no longer available for selection by others.

#### Group Based-Chat

Nakama’s group-based chat feature was implemented to allow users to communicate as peers. The users are required to consent to follow community rules for chatting. Users also receive notification reading, “reminder to adhere to the community guidelines,” that appeared for 15 s prior to ever entering the chatroom. To protect identities, only Avatars and nicknames appear in chats. Though Emoji use is allowed, we disabled features for making voice or video calls and for uploading media within the chat forum based on stakeholder feedback.

#### Messaging for Behavior Change

Informed by lessons around normative messaging for behavior change, our team developed and implemented culturally- and age-appropriate relevant messages to the implementation context (Table 2). These messages are displayed when particular criteria for the user relative to the group as illustrated in Table 2.

## DISCUSSION

ART adherence is central to effective HIV treatment, but AYA have high rates of virologic failure, virologic rebound after initial suppression, and attrition from HIV care. Complications from perinatal HIV infection and unique developmental features of adolescence and young adulthood make daily medication adherence even more challenging in this population. We utilized the IDEAS framework (41) to build on experience from the *TreatYourSelf* application (32–34) to develop *PEERNaija*, a theory-driven mHealth intervention aimed at improving medication adherence for AYA-HIV in LMIC settings. Grounded in Social Cognitive Theory, behavioral economics and principles of contingency management, *PEERNaija* employs several behavior change techniques to address common individual, environmental, and structural barriers to medication adherence identified by AYA-HIV including forgetfulness (9) and poor executive functioning (5, 8, 11), poor social support (5, 12–15), and nascent financial autonomy (5) making some clinic-based interventions less desirable. As its name suggests, *PEERNaija* also uniquely leverages the importance of peer relationships among AYA to incentivize medication adherence.

Two-way medication reminders are a critical component of this intervention. Indeed, while data suggest that daily, interactive text messages may be effective for AYA-HIV, such data are limited in LMIC and are limited primarily to SMS-based reminders (22, 26). In addition, further contextualization of the barriers to ART adherence for AYA-HIV suggest that medication reminders alone would leave other important barriers unaddressed. To our knowledge, despite the robust field of behavioral economics, there is very limited data exploring the role of social incentivization as a behavioral change technique. One pilot SMS-based intervention for AYA-HIV in Uganda found that youth who received information about their adherence scores relative to their peers showed improved adherence relative to standard of care, highlighting the promise of this approach (30). However, our app-based approach allows for versatility to adopt a multifaceted approach to behavior change, while also enabling the provider team to escalate outreach based on information collected by the app.

We have integrated several gamification approaches, described by Cugelman (72), to motivate both the adherence behavior itself and engagement with the application-based intervention. Despite the promise of mHealth-based approaches, application attrition and habituation are important obstacles (90). As such, we employed goal setting (leaderboard/levels and progress bar), capacity to overcome challenges (achievements and badges), provision of feedback on performance (leaderboard/levels, progress bar, and messaging for behavior change), reinforcement of behavior goals (leaderboard/levels, progress bar, and messaging for behavior change), and comparison of progress toward behavior goals (leaderboard/levels and progress bar). Fun and playfulness are promoted through the use of avatars and a point-based reward system. Social connectivity is another gamification approach that may be of particular importance for AYA-HIV. Lack of social support is a common obstacle to adherence, and fear of stigma and unwanted disclosure can further promote social exclusion and isolation from other potential sources of support (5, 12–15). *PEERNaija* provides a virtual, anonymous, peer-based social support group that may address an important need for social support, along with social accountability. This is especially important in a region where limited clinic-based outreach and largely unavailable adolescent-focused clinical services threaten adherence (9).

As a next step, we plan to gather detailed user feedback, conduct formal usability, acceptability, and feasibility testing of *PEERNaija* with AYA-HIV in Nigeria. We plan to evaluate this intervention among AYA-HIV in Nigeria using a pilot randomized controlled trial design. This pilot trial will focus on feasibility and acceptability, but will also allow us to identify characteristics of high and/or low utilization of various app components. Our study protocol also calls for direct outreach (phone then escalating to home-based outreach as needed) among participants who appear to be non-adherent to ART during our ecological assessments of adherence. We will also gather important information on potential implementation barriers such as cost (from the patient and provider perspectives), time, human resource, and structural needs (from the health system and patient perspectives) by tallying intervention costs and conducting FGDs with stakeholder groups at study conclusion. While we do not anticipate that this approach will be a “silver bullet” for all AYA-HIV, this work will provide important data for the potential role for a gamified smartphone application to deliver a multifaceted adherence interventions, along with essential peer support for vulnerable AYA-HIV in LMIC.

## Figures and Tables

**FIGURE 3 | (3.1–3.8):**
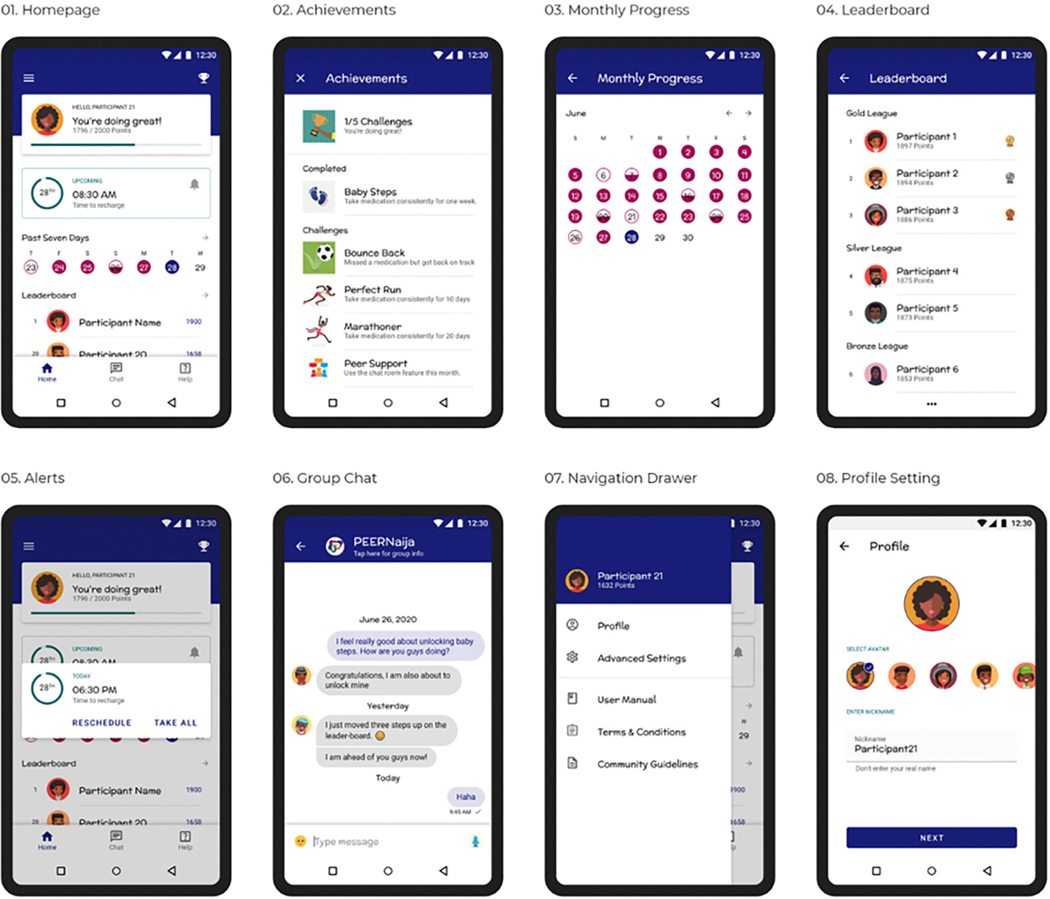
*PEERNaija* application user interfaces.

**TABLE 1 |:** Summary of behavior change techniques utilized to deliver intervention components in *PEERNaija* that address key adherence obstacles.

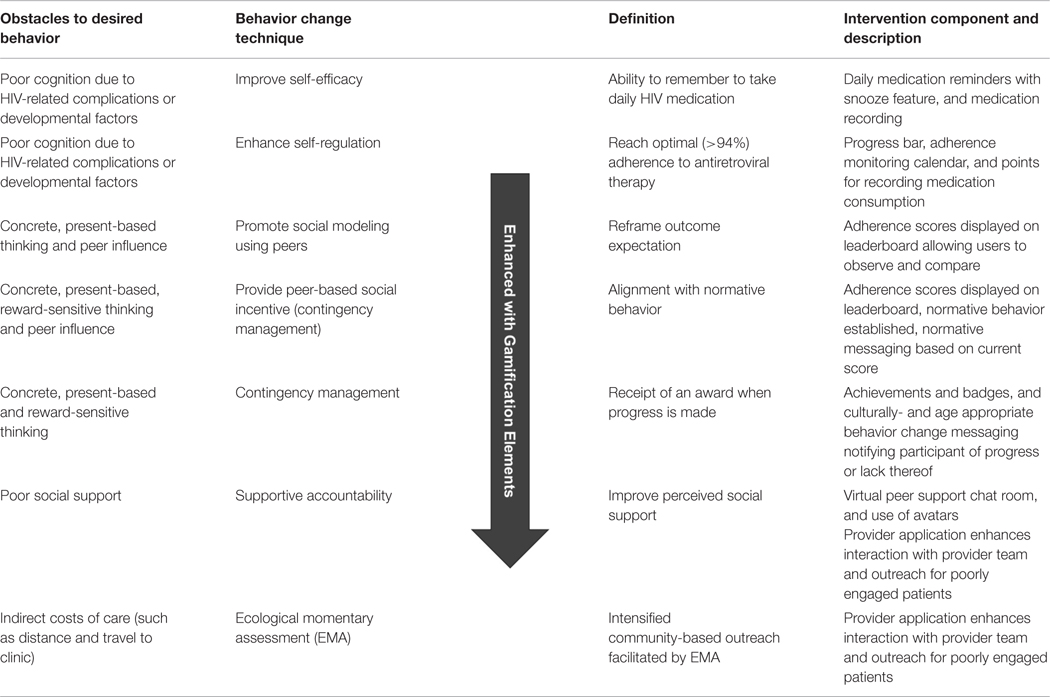

**TABLE 2 |:** Sample achievement and normative-based messages used in *PEERNaija*.

Reminder or achievement	Trigger	Sample message(s)

Daily medication reminder	Appears 30 min before the user’s scheduled medication time, and every 30 min for the next 4 h if the medication is not recorded	“The time don reach” “Time to recharge” “How you dey?” “You don chop?”
All doses taken	Appears when the user’s final medication of the day is taken	“You finish am”
No medications taken	Appears as the window for taking the medication has expired	“You no get am well”
Some doses taken	Appears as the window for taking the medication has expired for those taking twice daily (or more) medications	“You can do beta”
Baby steps	Appears after the user consistently takes medication for 5 days	“Small small”
Perfect run	Appears after the user consistently takes medication for 10 days	“You no dey play” “Nawaz Oh! Well done”
Marathoner	Appears after the user consistently takes medication for 20 days	“On fire”
Bounce Back	Appears after the user forgets to take a dose, but takes medication for 3 consecutive days following	“Welcome back”
Most improved	Achieved by the user with the most increased number of points from the previous month. Appear at the end of the months	“E don beta for you” “You dey do beta”

Normative condition	Trigger	Sample messages

Normative messages for optimal adherence (or above the norm)	Appears after adherence is >94% for 10 consecutive days	“Hey [username], your score don reach X%. Maintain the vibe!”
Normative Messages for Suboptimal/Poor Adherence (or below the norm)	Appears after adherence is <95% for 10 consecutive days	“You dey try. [Username], your paddy deem for PEERNaija don reach 95% but you dey Y%. No lose guard o”
